# Editorial: Databases and nutrition, volume II

**DOI:** 10.3389/fnut.2023.1307370

**Published:** 2023-11-22

**Authors:** Alessandra Durazzo, Igor Pravst, Massimo Lucarini

**Affiliations:** ^1^CREA-Research Centre for Food and Nutrition, Rome, Italy; ^2^Nutrition Institute, Ljubljana, Slovenia

**Keywords:** food data, food groups, nutrients, natural substances, dietary supplements, classification, categorization, food composition databases

## Introduction

This Research Topic is dedicated to covering high-level aspects of “Databases and Nutrition” from a global and interdisciplinary perspective and interoperability as a tool toward improved populational health. Studies that examine the relationship between diet and health have led to increased interest in nutrients and other biologically active constituents in food, and data on these and other compounds are increasingly required in the database system.

Detailed knowledge of the presence and levels of nutrients and bioactive components in food is the basis for data storage and structuration into databases.

Databases around food and nutrition represent fundamental resources and tools in many fields, such as nutrition, food science, public health, and healthcare, by supporting human research studies, policymaking, and consumer education.

The development of databases, based on foods and beverages consumed by the population, is based on efforts dedicated to measuring, collecting, and integrating data with information and detailed documentation on food and methodologies, etc., taking into account the current needs and demands, i.e., environmental aspects, lifestyle changes, and global market trade.

A lot of efforts have been made to harmonize food nutritional databases and repositories throughout worldwide projects and networks, leading to standard methodologies and guidelines, and also to promote applications of food classification and description systems and advances in systems for ontology alignment.

The standardization, harmonization, and FAIRization of data are being reached throughout automatized technologies, innovative systems, and digital tools for organizing and exploiting food data into various applications. Integrating and linking information and data from different sources, i.e., food, environmental, nutrition, and health ones, lead to the development of a comprehensive, multidimensional resource -based on integrating modeling, a digital platform, and cloud space at the multidimensional and multisource level- that can be used across disciplines.

Thirteen articles are published in the collection of articles under the Research Topic “*Databases and Nutrition - Volume 2*”.

Ahmed et al. presented the Food Label Information Program (FLIP), a comprehensive data approach for the evaluation of the Canadian food supply, and the latest methods used in the development of this database. Ferraz de Arruda et al. discussed vegetable oils as a case study of food composition databases in the era of big data.

Gilbert et al. presented an algorithm-based mapping of products in a Canadian branded food and beverage database to their equivalents in Health Canada's Canadian Nutrient File. The study of Balakrishna et al. addressed classifications of food items for health requirements and nutrition guidelines using Gaussian mixture models. The study by Liu et al. presented a novel food-components-target-function (FCTF) evaluation and prediction model for food efficacy based on association rule mining. Endaltseva et al. used an eater-oriented knowledge framework for reducing salt and dietary sodium intake and reviewed and presented an interdisciplinary documentary base of dietary sodium consumption factors.

It is also worth mentioning the study of Malcomson et al., which presented the operationalization of a standardized scoring system to assess adherence to the World Cancer Research Fund and American Institute for Cancer Research cancer prevention recommendations in the UK biobank.

Vlassopoulos et al. reported the performance of Nutri-Score in branded foods in Greece. Building on food composition data, Hribar et al. presented a validation of the food frequency questionnaire for the assessment of dietary vitamin D intake. The investigation by Shabnam focused on the assessment of non-linearity in the calorie–income relationship in Pakistan.

Davison et al. showed how lower energy-adjusted nutrient intakes occur among food energy under-reporters with poor mental health. Another study by Tang et al. focused on the intake of dietary fiber and femoral bone mineral density among middle-aged and older US adults from a cross-sectional study of the National Health and Nutrition Examination Survey 2013–2014.

Mognard et al. reviewed and explored “Eating Out”, spatiality, temporality, and sociality, and presented a database for China, Indonesia, Japan, Malaysia, Singapore, and France.

This collection of research articles published as part of the Research Topic collection presented not only innovative approaches for the collection and management of big data in the area of nutrition but also demonstrated the use of such data for progress in nutrition research. The collection also demonstrated several opportunities that should be addressed with future studies. In the past, essential nutrients were the primary target for most nutrient and food datasets, with the result that databases have generally lacked detailed information about other food constituents, including bioactives and other components, and the effects of different processing techniques on their content in the resulting foods. Furthermore, the public health challenges of the highly populated and industrialized environment also highlight that other components in foods, including contaminants, should be taken into account.

## Author contributions

AD: Writing—original draft. IP: Writing—original draft. ML: Writing—original draft.

